# A Controlled Antibiotic Release System for the Development of Single-Application Otitis Externa Therapeutics

**DOI:** 10.3390/gels3020019

**Published:** 2017-05-17

**Authors:** Bogdan A. Serban, Kristian T. Stipe, Jeremy B. Alverson, Erik R. Johnston, Nigel D. Priestley, Monica A. Serban

**Affiliations:** 1 Materials Science Program, University of Montana, Missoula, MT 59812, USA; bogdan.serban@mso.umt.edu (B.A.S.); erik.johnston@umconnect.umt.edu (E.R.J.); 2 Interdisciplinary Studies Program, University of Montana, Missoula, MT 59812, USA; kristian.stipe@umontana.edu; 3 Department of Chemistry and Biochemistry, University of Montana, Missoula, MT 59812, USA; jeremy.alverson@umconnect.umt.edu (J.B.A.); nigel.priestley@mso.umt.edu (N.D.P.); 4 Department of Biomedical and Pharmaceutical Sciences, University of Montana, Missoula, MT 59812, USA

**Keywords:** thixotropic, hydrogel, drug release, antibacterial, otic

## Abstract

Ear infections are a commonly-occurring problem that can affect people of all ages. Treatment of these pathologies usually includes the administration of topical or systemic antibiotics, depending on the location of the infection. In this context, we sought to address the feasibility of a single-application slow-releasing therapeutic formulation of an antibiotic for the treatment of otitis externa. Thixotropic hydrogels, which are gels under static conditions but liquefy when shaken, were tested for their ability to act as drug controlled release systems and inhibit *Pseudomonas aeruginosa* and *Staphylococcus aureus*, the predominant bacterial strains associated with outer ear infections. Our overall proof of concept, including in vitro evaluations reflective of therapeutic ease of administration, formulation stability, cytocompatibility assessment, antibacterial efficacy, and formulation lifespan, indicate that these thixotropic materials have strong potential for development as otic treatment products.

## 1. Introduction

Ear infections are common pathological conditions caused by bacterial, viral, or fungal agents [1,2]. Although the ear might seem anatomically simple, in reality it is a complex organ that includes external, middle, and inner structures [3]. The outer ear includes the pinna, the external ear auditory canal, and the tympanic membrane (the eardrum). Its role is to funnel sound to the middle ear, which is comprised of a system of small bones (ossicles) that vibrate under the stimulation of the acoustic waves. Signals originating in the middle ear then stimulate the structures of the inner ear (cochlea and labyrinth), which sends information on balance and head position to the central nervous system. The inner ear communicates with the nasal cavity thought the Eustachian (auditory) tube that controls the pressure in the middle ear and drains accumulated secretions.

Infections can affect all three structures of the ear, however, with different prevalence and symptomology [4]. Middle ear infections, or otitis media (OM), predominantly affect the pediatric population [5,6]. Its symptoms include pain, diminished hearing and, in severe cases, or if left untreated, OM can lead to hearing loss and other associated developmental sequelae (speech, communication) [5]. Clinical studies showed that five out of six children experience OM by the time they are three years old [7]. Due to the tympanic membrane barrier, OM treatment typically requires systemic administration of antibiotics [8]. Recently, therapeutics have been successfully administered to the middle ear through intratympanic injection or perfusion [9,10]. Outer ear infections, also known as otitis externa (OE), or Swimmer’s Ear, can affect a larger demographic that includes children, adolescents, and adults [11]. In 2007 in the United States, approximately 2.4 million health care visits were diagnosed with OE, with ~35% pediatric patients and ~53% adults [12]. Although OE can have various etiologies, in North America bacterial infections (typically attributable to *Pseudomonas aeruginosa* or *Staphylococcus aureus*) account for approximately 98% of all cases [13,14]. OE treatment regimes prescribe topical analgesics, locally-acidifying solutions (2% acetic acid), and/or antibiotic ear drops (gentamycin sulfate 0.3%, ofloxacin 0.3%, etc.) [15]. Topical antibiotic drops are preferred to their oral counterparts as the therapeutic is delivered directly to the infected tissue. However, they require multiple daily applications over 7–10 days, and studies show that only 40% of patients who self-medicate do so appropriately, with the effectiveness of the therapy increasing when someone else other than the patient applies the drops [16,17].

In this context, an effective, one-time application of a slow releasing drug system that would eliminate the need for multiple applications would bring significant benefits to current OE treatments. The data presented herein is associated with the initial, in vitro evaluation of a simple, dynamically cross-linked tetraethyl orthosilicate (TEOS)-based hydrogel controlled release system. We targeted the development of a thixotropic, easy to administer formulation with antibacterial efficacy as the foundation for further in vivo development of a one-time application therapeutic primarily intended for OE, with potential for intratympanic treatment of OM. Development parameters specifically addressed in this study were ease of administration, formulation stability, cytocompatibility assessment, antibacterial efficacy, and formulation lifespan.

## 2. Results

### 2.1. Hydrogel Formation

**Figure 1:**
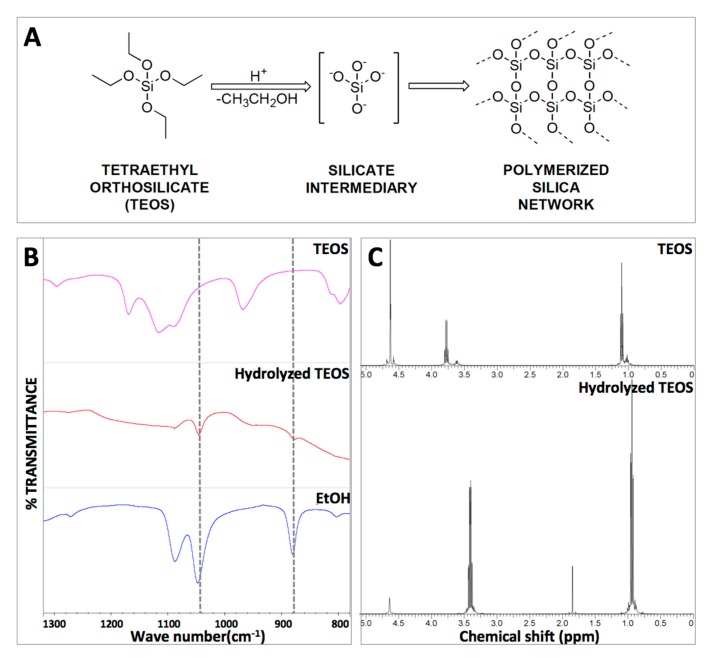
Tetraethyl orthosilicate (TEOS) hydrolysis. (**A**) Reaction scheme for the formation of the SiO_2_ network due to TEOS hydrolysis; (**B**) Fourier-transformed infrared spectroscopy (FTIR) monitoring of TEOS hydrolysis indicating the apparition of the ethanol peak—a side product of the TEOS hydrolysis reaction; and (**C**) Proton nuclear magnetic resonance (^1^H-NMR) analysis and confirmation of TEOS hydrolysis. The upper spectrum corresponds to TEOS, while the bottom spectrum shows a shift in the –CH_2_– (methylene) peak from 3.8 to 3.4 ppm and –CH_3_ (methyl) peak from 1.1 to 0.9 ppm, indicative of hydrolysis. The 2.0 ppm peak in the hydrolyzed TEOS spectrum corresponds to the methyl groups of the acetic acid used for hydrolysis.

**Figure 2:**
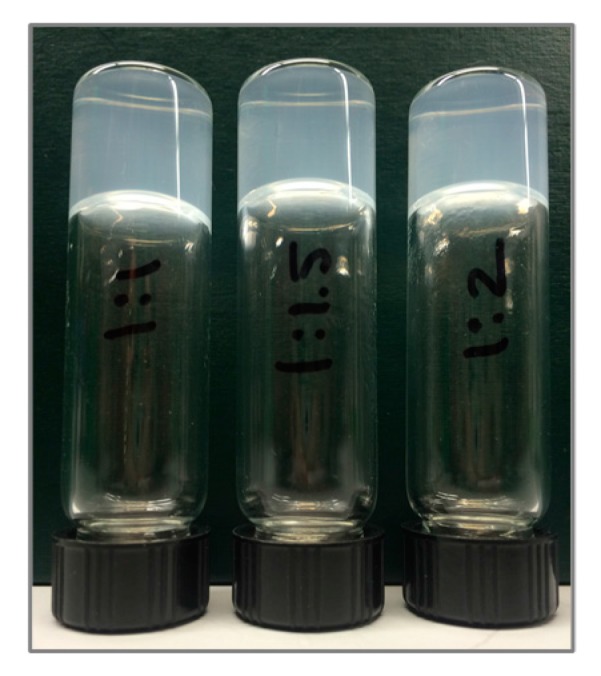
Physical appearance and optical clarity of thixogels.

In the framework of an easy to apply therapeutic formulation, we sought to base our system on a thixotropic material that liquefies under shear stress or agitation (during application) but becomes a gel under static conditions (upon application). To this end, TEOS was hydrolyzed under acid conditions to yield the transient silicate that serves as a hydrogel precursor or polymerization building block (Figure 1A). In this process, ethanol is formed as a secondary reaction product and its presence in the reaction mixture is indicative of hydrolysis. The formation of hydrolyzed TEOS (hTEOS) was monitored via Fourier-transformed infrared spectroscopy (FTIR) and proton nuclear magnetic resonance (^1^H-NMR), with both methods showing the peaks corresponding to the formation of ethanol. Specifically, the FTIR data shows the presence of ethanol specific peaks at 880 cm^−1^ (C–C–O stretch) and 1045–1088 cm^−1^ (symmetric and asymmetric C–O stretch, respectively) (Figure 1B). The ^1^H-NMR analysis confirmed the formation of ethanol via TEOS hydrolysis by the shift of the –CH_2_– (methylene) peak from 3.8 to 3.4 ppm and –CH_3_ (methyl) peak from 1.1 to 0.9 ppm (the 2.0 ppm peak in the hydrolyzed TEOS spectrum corresponds to the methyl groups of the acetic acid used for hydrolysis) (Figure 1C). For hydrogel preparation, hTEOS was mixed with diH_2_O in different ratios (Table 1) and allowed to gel (gelation was typically observed within minutes). All three formulations (TXL, TXM, and TXH, defined in Table 1) yielded optically-clear hydrogels (Figure 2).

### 2.2. Hydrogel Rheology

**Figure 3:**
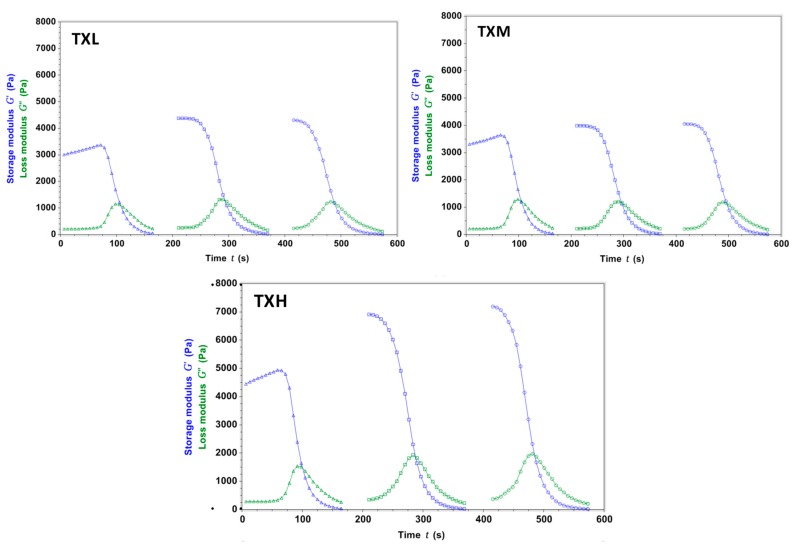
Rheological evaluation of hydrogel thixotropy during three stress cycles. All three formulations show stress-dependent gel-sol transitions. After the first cycle, for all formulations, the storage modulus (G′) values were higher, most likely due to polymeric network consolidation through solvent exclusion.

**Figure 4:**
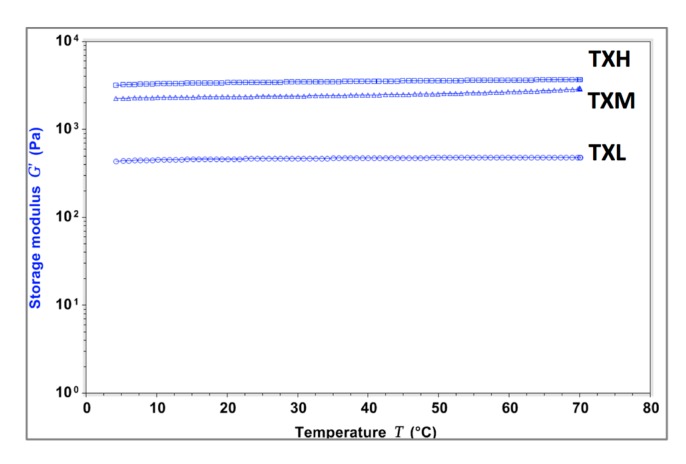
Evaluation of the temperature dependent behavior of the thixogels. A slight temperature dependence (approximately 10% increase in G′) is observed at temperatures above 60 °C, probably due to solvent loss.

**Figure 5:**
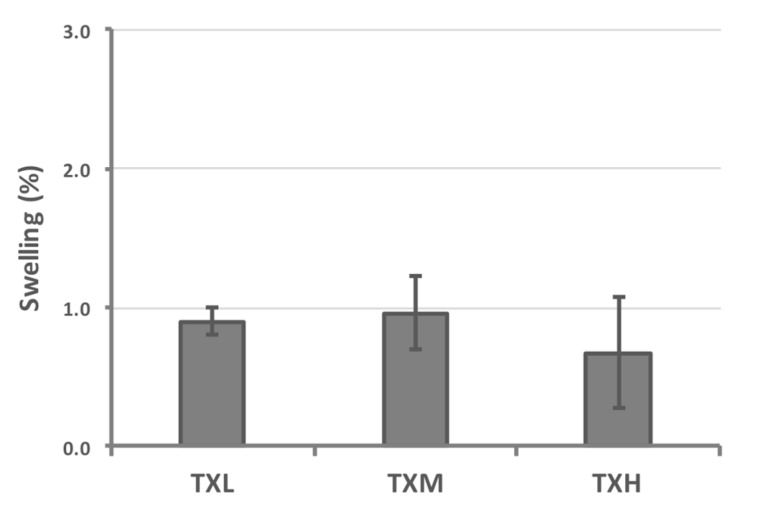
Evaluation of the swelling behavior of thixogels in aqueous environments, indicating that the hydrogels minimally change their volumes (approximately 1%) in the presence of physiological fluids (no statistically significant differences were noted between the three formulations).

The thixotropic properties of the three different hydrogel formulations were investigated rheologically. All three systems (TXH, TXM, and TXL) transitioned between gel-sol states as a function of stress, and this behavior was consistent throughout the randomly-chosen three testing cycles (Figure 3). The stiffness values of the thixogels were in the 3–5 kPa range and correlated with the amount of TEOS in the mixture as illustrated by the storage modulus (G′) values (G′_TXL_ < G′_TXM_ < G′_TXH_). For all three formulations, the G’ increased (4.2–7.2 Pa range) after the first shear cycle, most likely due to the consolidation of the polymeric network though water exclusion. In the context of the final targeted application (otic therapeutic), we tested the temperature dependent behavior of these materials to better understand their behavior at their potential storage and shipment conditions (refrigeration, room temperature), physiological conditions (37 °C), and elevated temperatures that might occur during typical transportation conditions (>40 °C) (Figure 4). The data indicate that TXL, TXM, and TXH are stable in the 4–60 °C range, with a ~10% increase in stiffness in the 60–70 °C (approximately 500 Pa) range most likely due to water loss. Next, the swelling behavior of these materials was investigated to better understand their behavior in moist or fluid filled environments. During the 30 min testing time, at 37 °C immersed in PBS, the hydrogels showed only a modest volume increase (approximately 1%) with no statistically significant difference between the three formulations (Figure 5 and Table 2). Overall the rheological characterization indicates that all three materials elicit thixotropy and are stable under physiological conditions.

### 2.3. Cellular Effects of Hydrogels

**Figure 6:**
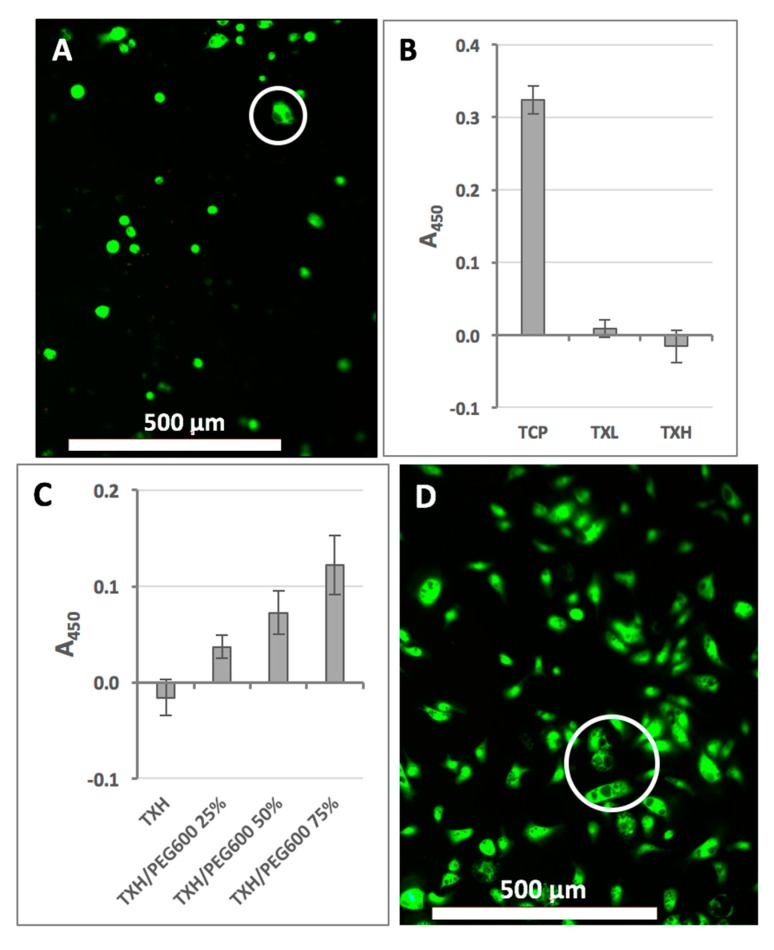
Thixogels cytocompatibility assessment. (**A**) LIVE/DEAD evaluation of cells on TXH indicating the presence of active intracellular esterases, intact cell membranes and some cytoplasmic vacuolation (circled); (**B**) evaluation of cellular metabolic activity via methyl tetrazolium salt (MTS) Cell-Titer assay, indicating reduced mitochondrial activity on TXL and TXH; (**C**) improvement of cellular metabolic activity through the addition on polyethylene glycol, molecular weight 600 Da (PEG600) to TXH; and (**D**) LIVE/DEAD evaluation of cells on TXH/PEG600 75% showing a more physiological spindle-like morphology with some cytoplasmic vacuolation (circled).

To evaluate the effect of hydrogels on cells, primary fibroblasts were employed and tested on the TXH formulation as it contained the highest amount of hTEOS (worst case scenario). When stained with calcein-AM and ethidium homodimer (LIVE/DEAD assay kit), the majority of cells elicited green fluorescence, typically indicative of cell viability (Figure 6A). Morphologically, cells were rounded, as commonly observed on matrices without cell attachment sites [18]. However, when assessed for nicotinamide adenine dinucleotide phosphate (NAD(P)H) oxido-reductase activity (Cell-Titer assay) cells on TXH showed no activity (Figure 6B) [19]. Cells cultured on TXL, however, elicited higher oxido-reductase activity compared to their TXH counterparts. We postulated that the observed metabolic rate reduction might be due to the cellular internalization of silica from the hydrogels, and that the addition of cytocompatible macromolecules to the formulation would alleviate this effect. To this end, TXH hydrogels were prepared with different amounts of a high molecular weight polyethylene glycols (PEGs). Specifically, from the available PEG polymers, we used polyethylene glycol, molecular weight 600 Da (PEG600) (25%, 50%, and 75%) as having a larger chain/molecular weight than polyethylene glycol, molecular weight 200 Da (PEG200). On PEG600-containing substrates, our data indicate that cells display a more physiological, spindle-like morphology and that their metabolic activity is significantly increased compared to the TXH control (Figure 6C). Cells on all formulations appeared to elicit cytoplasmic vacuolation, suggesting that the cellular effects observed are likely caused by the uptake of silica rather than cell membrane disruption, as substantiated by the LIVE/DEAD data (Figure 6D). Overall, our data indicate that the cytocompatibility of the thixogels can be tailored either by modulating the amount of hTEOS in the gels (resulting in softer materials) or by blending in large molecular weight cytocompatible macromolecules, such as PEG.

### 2.4. Controlled Release Evaluation

**Figure 7:**
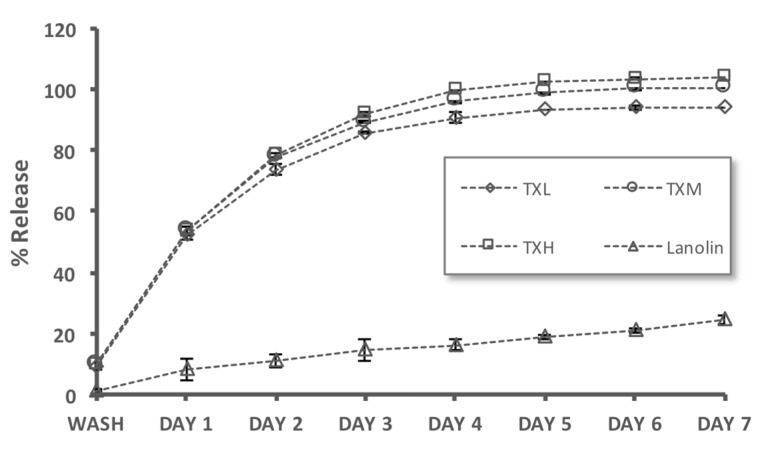
Evaluation of the controlled release capabilities of the thixogels by using fluorescein as a model drug. Lanolin—a compounding wax used for otic ointments—was used as a control. All three thixogels elicited controlled release properties for seven days.

We next investigated the controlled release capabilities of the thixogels by using fluorescein (MW = 332 Da) as a model drug. Fluorescein release is easy to detect spectroscopically and the molecule has comparable hydrophobicity and molecular weight to gentamycin (mixture of gentamycin c1, c1a, and c2, MW ~450 Da), a commonly-used antibiotic for OE treatment [15]. As a slow-release control we used lanolin (a pharmaceutical ointment base) loaded with fluorescein. All three thixogels elicited controlled release properties for seven days, with a faster release rate in days 1–3, then slowly plateauing between days 4–7 (Figure 7). The lanolin control release rate was much slower, approximately linear, and within seven days only ~20% of the fluorescein was released.

**Figure 8:**
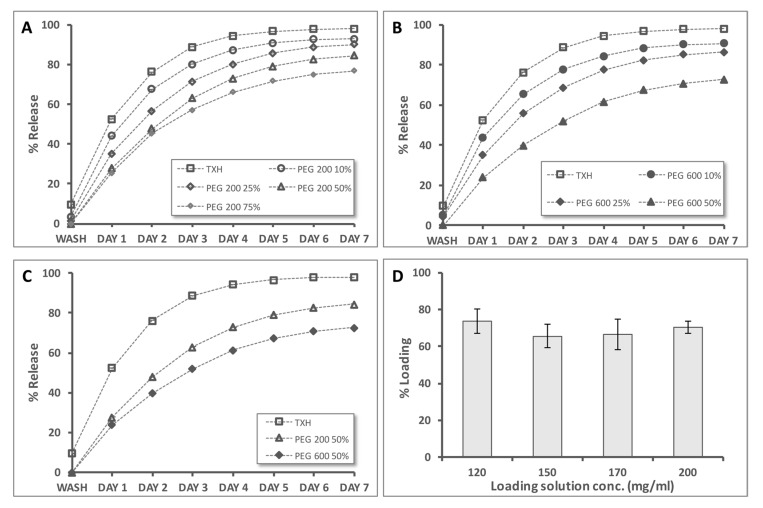
Fluorescein release from thixogels indicating the controlled release capabilities of the hydrogels. (**A**) Evaluation of the effects of PEG200 addition, in different amounts, on the fluorescein release properties, compared to TXH; (**B**) evaluation of the effects of PEG600 addition, in different amounts, on the fluorescein release properties, compared to TXH; (**C**) comparison of TXH, TXH + PEG200 50%, and TXH + PEG600 50% release rates indicating that longer PEG chains decrease the release rates; and (**D**) assessment of the loading capacity of the thixogels with four different concentrations of fluorescein, indicating a consistent loading efficiency of ~70%.

In the context of the cell assay data, we next evaluated the impact of blending in PEGs of different molecular weights/chain lengths and concentrations on the release rates (Figure 8).

Compared to the TXH samples, PEG200 containing samples released fluorescein at slower rates, with only 80% of the molecule released by day 7 from the TXH/PEG200 75% formulation (Figure 8A). Similar trends were obtained for the PEG600 containing thixogel formulations (Figure 8B). When assessing the effect of PEG molecular weight on the drug release rates, it appears that longer polymer chains (PEG600) are more effective in decreasing the release rates compared to their shorter chain counterpart (PEG200) (Figure 8C). For the TXH hydrogels, the drug loading efficiency was in the 65–75% range for fluorescein solution concentrations (between 120–200 mg/mL) (Figure 8D). Overall, these experiments indicate that thixogels are suitable systems for control release applications. Moreover, the release rates can be tailored through several mechanisms: a—adjustment of the hTEOS ratio; b—addition of macromolecules, such as PEG; and c—variation of the polymeric chain length/molecular weight of the added macromolecule.

### 2.5. Antibacterial Activity

**Figure 9:**
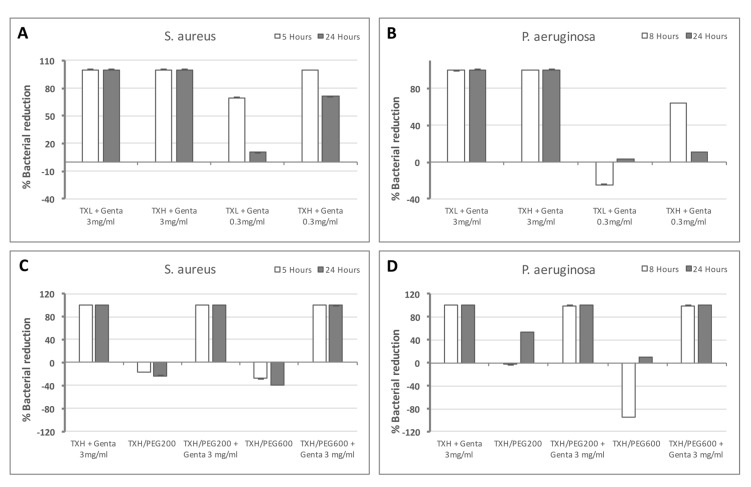
Evaluation of the antibacterial activity of thixogels. (**A**) Evaluation of the effect of TEOS amounts (TXL versus TXH) on *S. aureus* growth; (**B**) evaluation of the effect of TEOS amounts (TXL versus TXH) on *P. aeruginosa* growth; (**C**) evaluation of TXH hydrogels with and without PEG on *S. aureus* growth; and (**D**) evaluation of TXH hydrogels with and without PEG on *P. aeruginosa* growth.

With the confirmation that the hydrogels are capable of controlled release of small molecules, we tested their efficacy as antibacterial delivery systems. For this, gentamycin-loaded hydrogels, with or without PEG, were tested with *S. aureus* and *P. aeruginosa*, the most common strains associated with OE (Figure 9). The data presented is normalized to TXL and TXH, respectively, and the gentamycin concentration used is similar to currently used otic drops (0.3% (*w/v*) or 3 mg/mL). We first looked at the effect of the hTEOS ratio on bacterial growth (Figure 9A,B). With *S. aureus* both TXL and TXH induced a 100% growth inhibition when loaded with 3 mg/mL gentamycin at five and 24 h (Figure 9A). When 10-times less gentamycin was used in the hydrogels (0.3 mg/mL), at five hours, TXL + 0.3 mg/mL gentamycin induced only ~70% growth inhibition, while at 24 h, the bacteriostatic effect decreased to ~10%. In contrast, at 5 h, TXH + 0.3 mg/mL gentamycin still induced 100% growth inhibition, while at 24 h this effect decreased to ~70% (Figure 9A). For *P. aeruginosa*, both TXL + 3 mg/mL gentamycin and TXH + 3 mg/mL gentamycin elicited effects similar to *S. aureus*, for both the stain doubling time (8 h) and at 24 h (Figure 9B). At lower gentamycin concentrations (0.3 mg/mL), TXL appears to promote bacterial growth (no antibacterial effect), while at 24 h, it reduced growth rates by ~4%. TXH + 0.3 mg/mL gentamycin induced a ~65% decrease in growth, while at 24 h this effect diminished to ~11% (Figure 9B). These results appear to indicate that loading of the hydrogels with amounts higher than 0.3 mg/mL is needed to reach and maintain the minimum inhibitory concentration (MIC) for the tested stains. The data is consistent with the different drug release rates observed for TXL and TXH (the TXH release rate is higher than TXL), with the doubling time effects observed with the lower gentamycin concentration for both bacterial stains indicative of a higher initial dose of antibiotic released by TXH. Next, we investigated the effect of the addition of PEG polymers on bacterial growth (Figure 9C,D). For *S. aureus*, all antibiotic-containing formulations (TXH + 3 mg/mL gentamycin, TXH/PEG200 + 3 mg/mL gentamycin, and TXH/PEG600 + 3 mg/mL gentamycin) induced a 100% bacterial reduction, both at *S. aureus* doubling time (5 h) and at 24 h (Figure 9C). In contrast, the TXH/PEG200 and TXH/PEG600 controls appeared to accelerate bacterial growth, indicating that the observed bacteriostatic effects are due to the release of the antibiotic and are not intrinsic to the thixogels. For *P. aeruginosa*, similarly to *S. aureus*, the antibiotic-containing formulations (TXH + 3 mg/mL gentamycin, TXH/PEG200 + 3 mg/mL gentamycin, and TXH/PEG600 + 3 mg/mL gentamycin) induced a 100% bacterial reduction, both at the strain’s doubling time (8 h) and at 24 h (Figure 9D). The TXH/PEG200 control shows a slight growth acceleration at 8 h, and a ~50% growth reduction at 24 h, while at 8 h TXH/PEG600 controls appear to accelerate bacterial growth, but at 24 h induces ~10% growth reduction. It is unclear why the two bacterial strains tested show different trends on the control gels. The *P. aeruginosa* effects seen with the PEG-containing controls might be due to an acute metabolic rate inhibition mechanism similar to that seen with our primary fibroblasts. Within this hypothesis, *S. aureus* might not internalize silica nanoparticles at all, or it might incorporate them at much slower rates, with effects not detectable within the maximum duration of our assays (24 h). Nevertheless, our data indicate that gentamycin loaded thixogels are effective as antibacterial systems, and that the addition of different molecular weight polymers, such as PEG, does not affect their bacteriostatic capabilities.

### 2.6. Hydrogel Dehydration Rates

**Figure 10:**
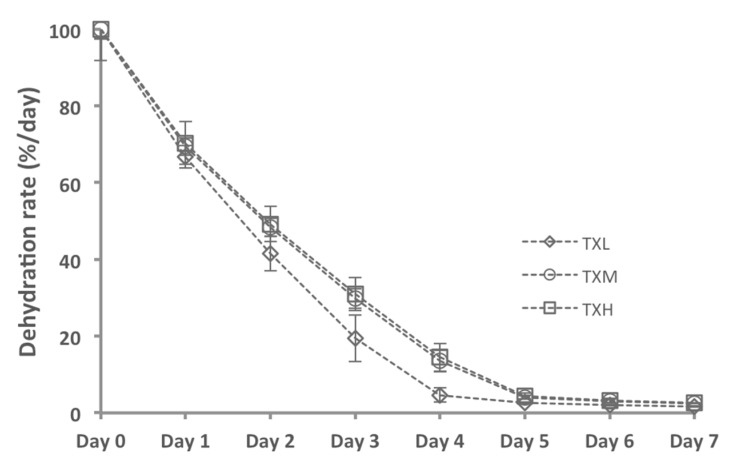
Evaluation of thixogel dehydration rates indicating that all formulations would gradually dry out to a small amount of dry material, and most likely would be naturally eliminated without causing hearing impairment.

To understand the lifespan of the hydrogels after application in the otic environment, we investigated the dehydration rates of these materials, as the most probable mechanism of gel dissipation. The physiological environment was mimicked by placing these gels under static conditions in a humidified oven set to 37 °C. Our data indicate that, under our test conditions, an aliquot of 100 μL thixogel undergoes dehydration within five days, reverting to a final dry mass of ~1.2 μg for THL and TXM and 1.24 μg for TXH (Figure 10 and Table 3). These data suggest that the thixogels would gradually dry out to a small amount of dry material, and would be naturally eliminated.

## 3. Discussion

The development of novel therapeutics is a complex process that targets the advancement of safe and effective compounds to commercialization. The use of well-characterized, regulatory agency-approved, cost-effective starting materials can significantly reduce the product development costs, the regulatory approval timeline, and is a practice commonly employed by industry for the expansion of their product pipeline [20]. Second, patient-centered pharmaceutical design heavily weighs on the success of a product, its acceptability, and medical outcome. Having the end-user of a therapeutic in mind early on in the development process can significantly speed up the commercialization of a product [21]. With all of the aforementioned considerations, this paper presents our approach to the development of a simple, easy to apply drug release system with potential to be developed into an otic product. TEOS has been widely characterized as a sol-gel transient system, and several of its applications as nanoparticle-based drug delivery vehicles have been reported. In the United States, TEOS is Food and Drug Administration (FDA)-approved as an indirect food contact substance/additive (demonstrated safe for their intended use), and silica-coated super paramagnetic iron oxide nanoparticles (SPION) recently received FDA approval as a biomedical photoacoustic contrast agent [22,23]. Additionally, TEOS-based therapeutics have already been moved to commercialization in Europe [24]. The data herein show that TEOS-based hydrogels can be formulated into an easy to apply, antibacterial system. These hydrogels would liquefy by simple shaking, making their application into the ear canal straightforward. Once in place, they would gel in situ and release the antibiotic at an effective therapeutic rate. As indicated by our data, the gels would maintain their volume and overall properties under physiological conditions. Given that TEOS hydrogels form through a polymerization mechanism that involves the formation of nanoparticles, the cellular effects due to the intrinsic nanoparticle uptake need to be addressed through further application-specific optimization [25,26]. Our first line of cytocompatibility testing shows that the cellular effects of these hydrogels can be modulated through the addition of high molecular weight biocompatible polymers, but further studies will be needed to fully understand the cytocompatibility requirements of external otic products.

## 4. Conclusions

This study presents the initial characterization steps required for the formulation of a target product profile for otic applications. It builds on comprehensive product development considerations that take into account, early on, the regulatory, scalability, and manufacturing implications. The data herein show preliminary findings pertaining to efficacy, safety, stability, and degradation. Further studies will specifically target the tailoring of hydrogels’ biocompatibility in organotypic models and will investigate their performance in preclinical studies. Overall, this study underlines the feasibility of these systems as single-application antibacterial otic therapeutics.

## 5. Materials and Methods

### 5.1. Materials

Tetraethyl orthosilicate (TEOS) and fluorescein were purchased from Sigma-Aldrich Chemical Co. (Milwaukee, WI, USA). Acetic acid (HOAc) was from EMD Millipore (Billerica, MA, USA), ammonium hydroxide (NH_4_OH) was from Fisher Chemical (Fair Lawn, NJ, USA), phosphate-buffered saline (PBS) was from ATCC (Manassas, VA, USA) and polyethylene glycol (PEG) with MW = 200 Da (PEG200), and MW = 600 Da (PEG600), respectively, were purchased from Alfa Aesar (Ward Hill, MA, USA). Gentamycin sulfate 600 IU/mg was from Alfa Aesar.

### 5.2. Analytical Instrumentation

Proton nuclear magnetic resonance (^1^H-NMR) spectral data were obtained using a Varian INOVA 400 at 400 MHz (Agilent Technologies, Palo Alto, CA, USA). Fourier-transformed infrared (FTIR) spectroscopic measurements were performed with a Nicolet™ iS™ 5N FT-NIR Spectrometer (Thermo Fisher Scientific, Waltham, MA, USA). Controlled release data was obtained with a FilterMax F5 microplate reader (Molecular Devices, Sunnyvale, CA, USA). Rheological data was acquired with a hybrid Discovery HR-2 Rheometer/Dynamic Mechanical Analyzer (TA Instruments, New Castle, DE, USA). Bacterial assay data was collected with a SpectraMax M4 plate reader.

### 5.3. Gel Formation

TEOS was hydrolyzed with 0.15 M HOAc for 1.5 h at a 1:9 (*v/v*) ratio. Hydrolyzed TEOS (hTEOS) was then combined with deionized water (diH_2_O) in different ratios (Table 1). The solutions were vortexed and the pH was adjusted to ~2 with 3.0 N HOAc. After 3 h, the pH was raised to 8.5 with 1.5 N NH_4_OH. All solutions formed gels when left unstirred overnight at room temperature. Gels were subsequently washed with diH_2_O to remove the residual ethanol (TEOS polymerization reaction by-product). For PEG containing hydrogel formulations, PEG solutions (PEG200, PEG600) were prepared in water at concentrations ranging from 10% to 75% (*v/v*). PEG solutions were mixed with hTEOS similarly to ratios shown in Table 1 (instead of diH_2_O, PEG solutions of different concentrations were used).

### 5.4. Controlled Release

Fluorescein (MW = 332.31 Da) was used as a hydrophobic drug model and was solubilized in 0.15 M NH_4_OH. Hydrogel aliquots prior to gelation (1.980 µL) were transferred to 4 mL glass vials containing 20 µL of 10 mg/mL fluorescein solution, to yield a total volume of 2 mL. The mixtures were gelled overnight at room temperature, and then washed with PBS twice to remove ethanol. After washes, 2 mL of PBS were added to each vial. The vials were then placed to 37 °C with no shaking. The release of fluorescein was monitored at 24 h intervals by assaying 100 µL PBS from each vial. The PBS was discarded and replaced with fresh aliquots daily, for each vial. For the controlled release from PEG-containing gels, samples were prepared as above, except that hTEOS was combined with PEG solutions instead of diH_2_O, in a 2:1 (*v/v*) ratio. Specifically, the PEG solutions used were: PEG200 of 10%, 25%, 50%, and 75% (*v/v*); PEG600 of 10%, 25%, and 50% (*v/v*).

### 5.5. Rheological Characterization

All hydrogels were characterized within the material’s pseudo-linear viscoelastic range with a 1.00 mm gap, at 20 ?C, unless otherwise specified. Oscillatory strain sweeps for thixotropy investigation were conducted with an 8 mm parallel plate geometry within a strain range of 1–250% and an angular frequency of 10 rad/s. The wait time between the cycles was 30 s. For temperature-dependent hydrogel behavior evaluation, samples were loaded onto the Peltier plate and a 20 mm parallel plate geometry (to provide a larger temperature exchange surface) was used for characterization. The hydrogel samples were equilibrated to 4 °C then subjected to a temperature ramp of 2 °C/min up to 70 °C, at a stain rate of 0.4% and an angular frequency of 10 rad/s. Swelling tests were performed with the 20 mm flat plate geometry and an active axial force control of 0.1 N. Hydrogels were loaded onto the Peltier plate and equilibrated at 37 °C for 6 min. PBS pre-warmed to 37 °C was then added and the gap distance between the plate and geometry was monitored for 30 min post addition. Initial gap heights varied from 1000 to 2000 µm depending on sample loading variations. All volumes were calculated assuming a perfect cylinder with a radius of 10 mm and a height measured by the rheometer.

### 5.6. Cell Assays

Primary adult normal dermal fibroblasts (HDF) (Lonza, Walkersville, MD, USA) were used to assess the cytocompatibility of the thixogels. Cells were seeded in a 24-well plate coated with 250 µL hydrogels (seeding density was 2 × 10^5^ cells/well in 500 µL) in serum-free fibroblast-conditioned media (Lonza, Walkersville, MD, USA) and incubated for 24 h at 37 °C/5% CO_2_. Cell viability was visually assessed with a LIVE/DEAD viability/cytotoxicity kit for mammalian cells. Cellular metabolic activity was quantified with a Cell-Titer 96 Aqueous One Solution Cell Proliferation Assay (Promega, Madison, WI, USA) and detected via absorbance at 450 nm with a microplate reader.

### 5.7. Bacterial Assays

Antibiotic susceptibility was assessed for *Staphylococcus aureus* 13709 and *Pseudomonas aeruginosa* 27853 using a broth microdilution protocol based on the Clinical and Laboratory Standards Institute (CLSI) guidelines. Testing was performed by first inoculating a shake flask containing 10 mL of Iso-sensitest media with 10 µL of glycerol stock for each strain. The cultures were then grown overnight for 8 h to the log phase at 37 °C with shaking at 225 rpm. The overnight cultures were then diluted in Iso-sensitest to approximately 6.7 × 10^5^ cfu/mL and 150 µL/well were added to 96-well test plates coated with 100 µL/well hydrogel with or without gentamycin. After incubation at 37 °C for either the strain-specific doubling time or 24 h, 100 µL aliquots from each test well were transferred to wells in clear bottom 96-well plates. A 10 µL sample of a 0.03% (*w/v*) aqueous solution of resazurin (fluorescent Alamar Blue assay) was then added to the wells and allowed to incubate at room temperature for 10 min. The fluorescent signal of the reduced dye was measured on a plate reader using excitation and emission wavelengths of 555 and 585 nm, respectively.

### 5.8. Statistical Analysis

Values, represented as the mean ± standard deviation (S.D.), were compared using the Student’s *t*-test (two-tailed, type 3) with *p* < 0.1 considered statistically significant.

## Figures and Tables

**Table 1:** Designation and formulation of thixogels.

Hydrogel	Designation	Formulation
		H_2_O:hTEOS (*v/v*)
Thixogel—low density SiO_2_ network	TXL	1:1
Thixogel—medium density SiO_2_ network	TXM	1:1.5
Thixogel—high density SiO_2_ network	TXH	1:2

**Table 2:** Summary of percent volume increase observed for the three thixogel formulations, indicating no statistical differences in the swelling behavior.

Hydrogel	Volume Increase Due to Swelling (%)	Standard Deviation (%)
TXL	0.90	0.10
TXM	0.96	0.26
TXH	0.67	0.40

**Table 3:** Quantification of the amount of dry weight per 100 µg of thixogel. All formulations appear to reduce to ~1% of their initial weight within five days, via water loss.

Hydrogel	Dry Weight/100 μg Hydrogel (in μg)	Standard Deviation (μg)
TXL	1.198	0.030
TXM	1.196	0.005
TXH	1.238	0.021
